# Drivers of Rising Prevalence in Major Motor Neurodegenerative Diseases: Temporal Trends in Sweden and France (2003–2022)

**DOI:** 10.1212/WNL.0000000000218072

**Published:** 2026-06-05

**Authors:** Octave Guinebretiere, Fen Yang, Dang Wei, Quentin Calonge, Yihan Hu, Karin Wirdefeldt, Caroline Ingre, Fredrik Piehl, Karin Modig, Weimin Ye, Maria Del Mar Amador, Stanley Durrleman, Gaelle Bruneteau, Celine Louapre, Jean-Christophe Corvol, Fang Fang, Thomas Nedelec

**Affiliations:** 1 Sorbonne University, Paris Brain Institute - ICM, CNRS, Inria, Inserm, AP-HP, Hôpital de la Pitié Salpêtrière, France;; 2 Sorbonne Université, INSERM, Institut Pierre Louis d'Epidémiologie et de Santé Publique, Equipe PEPITES, AP-HP, Hôpital Pitié Salpêtrière, Département de Santé Publique, Centre de Pharmaco, France;; 3 Institute of Environmental Medicine, Karolinska Institutet, Stockholm, Sweden;; 4 AP-HP, Epilepsy Unit, Center of Reference for Rare Epilepsies, ERN EpiCARE, Pitié-Salpêtrière Hospital, DMU Neurosciences, Paris, France;; 5 Department of Medical Epidemiology and Biostatistics, Karolinska Institutet, Stockholm, Sweden;; 6 Department of Clinical Neuroscience, Karolinska Institutet, Stockholm, Sweden;; 7 Department of Neurology, Karolinska University Hospital, Stockholm, Sweden;; 8 Neuroimmunology Unit, Center for Molecular Medicine, Karolinska Institutet, Stockholm, Sweden;; 9 Paris ALS Center, Department of Neurology, Hôpital de la Pitié-Salpêtrière, Assistance Publique-Hôpitaux de Paris (AP-HP), DMU Neurosciences, Paris, France; and; 10 Department of Neurology, Hôpital de la Pitié Salpêtrière, Assistance Publique Hôpitaux de Paris (APHP), DMU Neurosciences, Paris, France.

## Abstract

**Background and Objectives:**

The prevalence of Parkinson disease (PD), multiple sclerosis (MS), and motor neuron diseases (MNDs) is rising globally. However, it is unclear to what degree this is related to an increase in incidence or to improved survival after diagnosis.

**Methods:**

We performed 2 nationwide, population-based, retrospective cohort studies, including all individuals living in Sweden between 2001 and 2016 and living in France between 2009 and 2022, respectively. Pooled mixed-effects regression models, with country as a random effect, were used to determine temporal trends in prevalence, crude and age-standardized and sex-standardized incidence, and age and life expectancy at diagnosis.

**Results:**

Annualized prevalence of PD, MS, and MNDs increased significantly between 2003 and 2022 in the pooled model (PD: prevalence ratio [PR] per year = 1.014, *p* < 0.001; MS: PR = 1.029, *p* < 0.001; MND: PR = 1.028, *p* < 0.001). While the crude incidence of both PD and MS remained nearly stable over time (PD: incidence rate ratio [IRR] per year = 0.998, *p* < 0.001; MS: IRR = 0.992, *p* < 0.001), the standardized incidence showed a more marked decrease for PD (IRR = 0.986, *p* < 0.001) while remaining almost unchanged for MS (IRR = 0.995, *p* < 0.001). For MNDs, both the crude and standardized incidence increased over time (crude IRR = 1.018, *p* < 0.001; standardized IRR = 1.008, *p* < 0.001). Life expectancy at diagnosis of PD increased between 2003 and 2013 (+0.95 months per calendar year, *p* < 0.001) and then decreased between 2013 and 2022 (−1.20 months, *p* = 0.002), while it increased significantly over the entire study period for MS (+2.35 months, *p* < 0.001) and MNDs (+0.34 months, *p* = 0.01).

**Discussion:**

These findings indicate that the rising MS prevalence is largely survival-driven and the rising MND prevalence reflects a true increase in incidence, whereas PD prevalence grows modestly, largely independent of incidence. Depending on the mechanism that drives prevalence, whether increased incidence reflecting changing risk factor exposures, improved survival due to therapeutic advances, or demographic aging of the population, inferences about underlying causes differ substantially between PD, MS, and MNDs, with direct implications for health care planning and etiologic research.

## Introduction

Neurodegenerative diseases (NDDs) with motor symptoms, such as Parkinson disease (PD), multiple sclerosis (MS), and motor neuron diseases (MNDs), pose significant public health challenges due to their progressive nature and profound impact on quality of life. Understanding and comparing the burden of these diseases, including incidence, prevalence, and mortality, is crucial for informing health care planning, resource allocation, and the development of targeted interventions.^1-4^ While the prevalence of these diseases seems to be increasing globally, the underlying reasons for this trend are not well understood and could vary between diseases.^5,6^ An increasing prevalence could be attributed to an increase in disease incidence in aging populations, as well as improvements in life expectancy due to better management of NDDs and general health conditions such as cardiovascular diseases or cancers.

To date, comprehensive studies across multiple NDDs leveraging nationwide data from single or multiple countries are rare.^7^ The use of data from multiple countries is especially relevant to better understand how population-specific factors such as environmental exposures, lifestyle, and health care system may contribute to differences in disease burden. In addition, differences in recording practices—standardized coding, update frequency, and diagnostic criteria in health registers—can significantly influence how patients with NDDs are detected and tracked, affecting the accuracy and comparability of epidemiologic data across countries.^8,9^ Using health registers from multiple countries may, therefore, enable a more robust analysis of temporal trends in disease burden, alleviating the concern that any observed trends are reflective of not only the true trends but also artifacts in population-specific recording or database biases.

To this end, we established a research collaboration between French and Swedish teams to investigate the prevalence, incidence, age at diagnosis, and mortality of PD, MNDs, and MS in the entire Swedish (2003–2016) and French (2011–2022) populations. By leveraging data from 2 countries with availability of national health registers and universal coverage, our aim was to provide a comprehensive overview of the temporal trends in the burden of these diseases and highlight any disparities or similarities that may exist between countries.

## Methods

### Data Sources

The Swedish Total Population Register contains demographic information (including the date and place of birth, sex, and civil status) of Swedish residents since 1968.^10^ The Swedish National Patient Register (SNPR) collects information (disease diagnosis and date) on specialized inpatient care since 1964 (nationwide since 1987) and on outpatient care since 2001.^11,12^ The Swedish Prescribed Drug Register,^12^ initiated in July 2005, collects information on all dispensed drug use, from either specialized care or primary care. The French National Health Data System (SNDS) is a comprehensive and extensive repository of health care information established in 2009 and incorporates data from 2 primary sources, the national hospital discharge database for inpatient care and the national health insurance claims information system for outpatient care, covering approximately 99% of the population of France. SNDS contains detailed information on all reimbursed prescriptions in both inpatient and outpatient services.

### Identification of Patients With NDDs and Study Design

In Sweden, patients with NDDs were identified from the SNPR,^13^ using information on the primary or a secondary diagnosis made at an inpatient or outpatient hospital visit, namely ICD-9 code 332A and ICD-10 code G20 for PD, ICD-9 code 340 and ICD-10 code G35 for MS, and ICD-9 code 335C and ICD-10 code G12.2 for MNDs (eTable 1). The ascertainment of these diseases using SNPR has been validated in previous studies, showing a positive predictive value of 70.8% for PD^14^ and 95% for MS.^15^ Although the validity of MND diagnosis in Sweden has not yet been evaluated, the SNPR is generally recognized for its high completeness and accuracy.^16^ In France, patients with PD, MNDs, or MS were identified using the SNDS with validated algorithms from the literature (eTable 1). In Sweden, the date of diagnosis was defined as the first time the patient received a diagnosis of the respective disease in either an inpatient or outpatient hospital visit. In France, the date of diagnosis was defined as the first time the patient met 1 criterion from the validated algorithm, either through a prescription for a specific treatment or the first related diagnosis. Age restriction was only applied to PD, that is, an age at diagnosis of 20 years or older. We performed 2 nationwide, population-based, retrospective cohort studies, including all individuals living in Sweden between 2001 and 2016 and living in France between 2009 and 2022, respectively, using data from the Swedish Total Population Register and the French National Institute of Statistics and Economic Studies in France, respectively.^17-19^

### Statistical Analysis

#### Washout Period

As we aimed to study incidence to explain prevalence trends, we used the first 2 years of the study period in each cohort as a washout period for PD and MNDs to exclude prevalent cases, that is, starting in 2003 in Sweden and 2011 in France. For MS, however, the situation is more complex because of the existence of primary progressive and benign forms that may not require frequent medical attention. Therefore, a more conservative approach with a longer washout period of 5 years was applied for MS to better capture true incident cases.

#### Trend in Prevalence

Annual prevalence of NDDs was estimated by dividing the number of patients with PD/MS/MNDs still alive by the size of reference population in each cohort,^17-19^ using December 31st of 2003–2016 for Sweden and January 1st of 2011–2022 for France as cutoff dates. Temporal trends in prevalence during the study period were assessed using prevalence ratios (PRs) with 95% CIs, derived from Poisson regression models assuming a linear relationship between prevalence and time.^20^

#### Trend in Incidence

Annual incidence rates of NDDs were defined as the number of newly diagnosed cases in each calendar year divided by the person-years of the reference population on December 31st of that year for Sweden and on January 1st of the next year for France and were expressed per 100,000 person-years. To adjust for the potential impact of demographic shifts on incidence rates, standardized incidence rates were computed using the age and sex distributions of the total population at the baseline year of the study period (2003 for Sweden and 2011 for France) and in 5-year age and sex strata. 95% CIs of the crude and standardized incidence rates were calculated under a Poisson distribution assumption and assuming a linear relationship between incidence and time. Because the coronavirus disease 2019 (COVID-19) pandemic likely affected MNDs more strongly, given that its care pathway often requires hospitalization, we decided to exclude the years 2020–2022 from the Poisson regression analysis, while retaining them in the incidence curves for transparency.

#### Life Expectancy at Diagnosis

Life expectancy at diagnosis was estimated using both disease-specific and general population mortality life tables. Life expectancy at diagnosis was estimated using the median age at diagnosis in the pooled sample of the 2 cohorts, that is, 75.6 years for PD, 42.4 years for MS, and 69.2 years for MNDs. Specifically, we constructed abridged life tables over successive 2-year periods to summarize mortality patterns across all age groups. We reported the yearly variation in life expectancy (in months), assuming a restricted cubic spline model relationship between life expectancy and time as the primary analysis. In a secondary analysis, because the linear assumption did not hold for PD, we fitted 2 separate linear models: one for the period 2003–2013 and another for 2013–2022.

#### Pooled Models

In addition to reporting country-specific results, we also used mixed-effects models (Poisson regression models for incidence and prevalence and regression models for age at diagnosis and life expectancy at diagnosis) to account for between-country heterogeneity and report pooled results between countries.

### Sensitivity Analyses

In the primary analyses, we assumed a linear relationship between calendar year and prevalence and incidence because visual inspection supported linearity and this approach allowed estimation of annual prevalence and incidence rate ratios (IRRs). Nonlinearity was explored in secondary analyses using restricted cubic spline models with 3–5 knots and compared using Akaike information criterion and Bayesian information criterion; a 3-knot model was retained, with knots at 2007 (Sweden-only period), 2013 (common period), and 2019 (France-only period). For life expectancy, nonlinear models were used in the primary analysis because trends were clearly nonlinear, with an additional secondary analysis approximating trends using 2 linear segments. A similar approach was applied to age at diagnosis because the linearity assumption was not met for MS. In a second sensitivity analysis, we further investigated potential between-country heterogeneity in temporal trends of incidence and disentangled effect of time and country, through fitting country-specific models during a period with greater overlap between countries (2009–2018).

### Standard Protocol Approvals, Registrations, and Patient Consents

The use of Swedish register data for this study was approved by the Swedish Ethical Review Authority (DNR 2024-05896-01). Owing to the register-based nature of this study, informed consent from individual participants was waived by this approval. The Swedish data are pseudonymized and thus subject to General Data Protection Regulation (GDPR) and cannot be shared openly. We accessed and processed data from the SNDS, as permanent access to the Caisse Nationale Assurance Maladie data portal was granted through the Institut National de Recherche en Informatique Appliqué affiliation of the ARAMIS team, in application of the provisions of Articles R. 1461–11 to R. 1461–17 of the French Public Health Code and the French data protection authority decision Commission Nationale Informatique Liberté (CNIL)-2016-316. As permanent users of the SNDS, the authors declared the study to the INRIA's SNDS registry and were exempted from Institutional Review Board approval. The data from the SNDS are governed by Articles R. 1461–11 to R. 1461–17 of the French Public Health Code and the French data protection authority decision CNIL-2016-316.

### Data Availability

The data for analyzing medications and health conditions are available for research purposes to qualified researchers on request to the Swedish National Board of Health and Welfare and Statistics Sweden, provided that they meet specific requirements. The data from the SNDS (France) are governed by Articles R. 1461–11 to R. 1461–17 of the French Public Health Code and the French data protection authority decision CNIL-2016-316 and cannot be shared publicly.

## Results

### Prevalence

In Sweden, 15,273 (185 per 100,000 individuals) patients with prevalent PD, 12,457 (151) with prevalent MS, and 825 (10.0) with prevalent MND were identified in 2003 and 20,839 (243) patients with prevalent PD, 19,864 (231) with prevalent MS, and 1,285 (15.0) with prevalent MND were identified in 2016. In France, 188,072 (290 per 100,000 individuals) patients with prevalent PD, 94,438 (145) with prevalent MS, and 5,796 (8.9) with prevalent MND were identified in 2011, and 227,594 (336) patients with prevalent PD, 130,676 (193) with prevalent MS, and 8,221 (12.1) with prevalent MND were identified in 2022 (Table 1). The number of individuals under each diagnosis is shown in eFigures 1–3.

**Table 1:** Characteristics of Prevalent and Incident Patients With MS, PD, and MNDs in France and Sweden (2003–2022)

	Parkinson disease		Multiple sclerosis		Motor neuron diseases	
	Sweden	France	Sweden	France	Sweden	France
Identification years	2003–2016	2011–2022	2003–2016	2011–2022	2003–2016	2011–2022
No. of cases (prevalent [crude ratio per 100,000 individuals]/incident [crude rate per 100,000 person-years])						
2003	15,273 (185)/2,244 (27.8)	—	12,457 (151)/1,178 (14.6)	—	825 (10.0)/305 (3.8)	—
2005	16,103 (194)/2,332 (28.8)	—	14,014 (169)/1,020 (12.6)	—	875 (10.6)/284 (3.5)	—
2007	17,132 (206)/2,503 (30.7)	—	15,307 (184)/966 (11.8)	—	938 (11.3)/298 (3.6)	—
2009	18,124 (216)/2,371 (28.9)	—	16,525 (197)/914 (11.1)	—	1,061 (12.7)/360 (4.4)	—
2011	19,109 (226)/2,544 (30.8)	188,072 (290)/22,253 (34.7)	17,701 (210)/976 (11.8)	94,438 (145)/5,295 (8.2)	1,115 (13.2)/355 (4.3)	5,796 (8.9)/1,940 (3.0)
2013	19,965 (234)/2,382 (28.6)	201,955 (308)/22,462 (34.3)	18,851 (221)/900 (10.8)	102,291 (156)/5,122 (7.9)	1,162 (13.6)/346 (4.1)	6,388 (9.7)/2,024 (3.1)
2015	20,839 (243)/2,454 (29.2)	214,079 (323)/23,551(35.6)	19,864 (231)/842 (10.0)	110,029 (166)/5,239 (7.9)	1,285 (15.0)/368 (4.4)	6,982 (10.5)/2,105 (3.2)
2017	—	222,623 (335)/23,003 (34.6)	—	117,180 (176)/5,048 (7.6)	—	7,552 (11.4)/2,209 (3.3)
2019	—	227,889 (340)/23,047 (34.4)	—	124,156 (185)/5,158 (7.7)	—	8,021 (12.0)/2,301 (3.4)
2021	—	227,594 (336)/23,014 (34.0)	—	130,676 (193)/5,292 (7.9)	—	8,221 (12.1)/2,257 (3.3)
Age at diagnosis, y, median (IQR)	75.1 (68.0–81.3)	76.1 (68.5–82.5)	43.2 (32.7–54.7)	41.6 (32.1–52.1)	69.4 (61.2–76.9)	69.1 (60.8–76.8)
Sex (incident), n (%)						
Men	19,995 (59.4%)	155,260 (56.6%)	4,145 (31.5%)	19,149 (31.0%)	2,648 (56.2%)	15,928 (56.7%)
Women	13,653 (40.6%)	119,219 (43.4%)	9,006 (68.5%)	42,650 (69.0%)	2,061 (43.8%)	12,184 (43.3%)

Abbreviations: IQR = interquartile range; MNDs = motor neuron diseases; MS = multiple sclerosis; PD = Parkinson disease.

**Figure 1:**
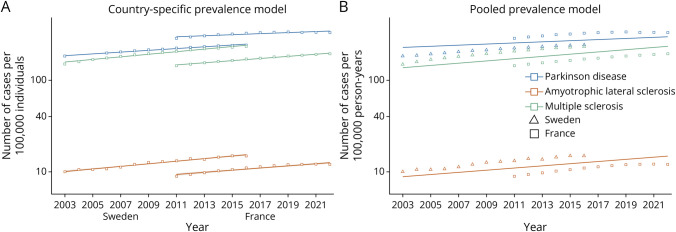
Country-Specific and Pooled Analysis of Secular Trends in Prevalence Assuming a Linear Relationship With Time in Sweden and France (2003–2022) Prevalence curves were estimated using single calendar year data points and the predictions of (A) the Poisson regression models from each country and (B) the population effect of the Poisson regression mixed-effects models. We assumed a linear relationship between time and incidence to compute prevalence ratios. Graph points were obtained from observed data.

For all 3 diseases, the prevalence increased over time in both countries (Figure 1, Table 2, eFigure 4). Pooled analyses of the 2 countries demonstrated an annual increase of 1.4% for PD, 2.9% for MS, and 2.8% for MND (all *p* < 0.001) (Table 2).

**Table 2:** Time Trends in Incidence and Prevalence

Time trend effect size (95% CI), *p* value	Crude			Standardized		
	Sweden	France	Pooled	Sweden	France	Pooled
Parkinson disease						
Prevalence (PR)	1.022 (1.021–1.024), *p* < 0.001	1.013 (1.009–1.017), *p* < 0.001	1.014 (1.014–1.014), *p* < 0.001	—	—	—
Incidence (IRR)	1.002 (0.999–1.005), *p* = 0.07	0.997 (0.996–0.998), *p* < 0.001	0.998 (0.997–0.999), *p* < 0.001	0.995 (0.992–0.997), *p* < 0.001	0.984 (0.983–0.985), *p* < 0.001	0.986 (0.985–0.987), *p* < 0.001
Multiple sclerosis						
Prevalence (PR)	1.033 (1.030–1.036), *p* < 0.001	1.028 (1.026–1.029), *p* < 0.001	1.029 (1.028–1.029), *p* < 0.001	—	—	—
Incidence (IRR)	0.985 (0.979–0.992), *p* < 0.001^a^	0.993 (0.991–0.995), *p* < 0.001	0.992 (0.990–0.994), *p* < 0.001	0.988 (0.982–0.994), *p* < 0.001^a^	0.996 (0.994–0.999), *p* = 0.002	0.995 (0.993–0.997), *p* < 0.001
Motor neuron diseases						
Prevalence (PR)	1.033 (1.030–1.036), *p* < 0.001	1.027 (1.023–1.032), *p* < 0.001	1.028 (1.026–1.030), *p* < 0.001	—	—	—
Incidence (IRR)	1.021 (1.014–1.029), *p* < 0.001	1.015 (1.010–1.021), *p* < 0.001^b^	1.018 (1.013–1.022), *p* < 0.001	1.014 (1.007–1.021), *p* = 0.005	1.005 (0.999–1.01), *p* = 0.078^b^	1.008 (1.004–1.012), *p* < 0.001

Abbreviations: COVID-19 = coronavirus disease 2019; IRR = incidence rate ratio; PR = prevalence ratio.

IRRs from country-specific regression models and mixed-effects models. We assumed a linear relationship between time and incidence.

a IRR computed for the 2006–2016 period.

b IRR computed for the 2011–2019 period (COVID-19 pandemic period excluded).

### Incidence

#### PD

**Figure 2:**
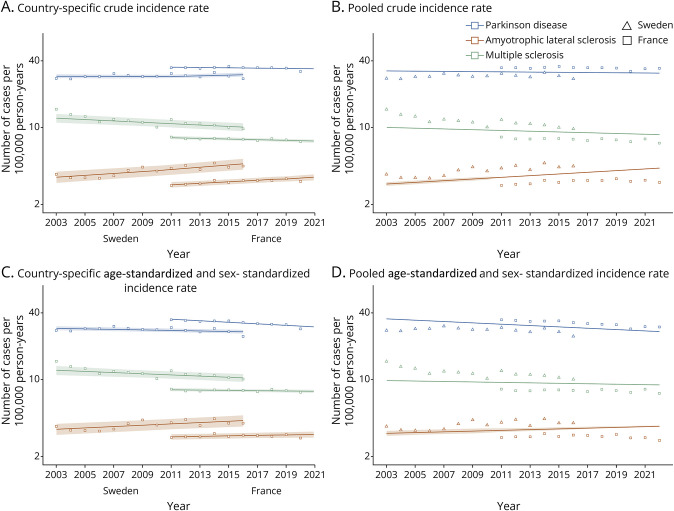
Country-Specific and Pooled Analysis of Secular Trends in Incidence Rates Assuming a Linear Relationship With Time in Sweden and France (2003–2022) Panel A: country-specific crude incidence rate. Panel B: pooled crude incidence rates. Panel C: country-specific age-standardized and sex-standardized incidence rate. Panel D: pooled age-standardized and sex-standardized incidence rate. Graph points were obtained from observed data. Incidence curves were estimated using single calendar year data points and Poisson regression. The incidence rate ratio refers to the average increase in incidence rate over 1 year.

Crude incidence rates were slightly higher in France (34.4 [95% CI 34.0–34.9]) than in Sweden (29.2 [28.0–30.3]), and the pattern was similar for standardized rates (France: 32.2 [31.8–32.6]; Sweden: 28.1 [26.9–29.3]), per 100,000 person-years (Figure 2). In Sweden, crude incidence rates remained relatively stable during the study period (IRR per year = 1.002, *p* = 0.07), whereas in France, a modest but significant decline was observed during the study period (IRR = 0.997, *p* < 0.001) (Figure 2, Table 2, eFigure 5). Mixed-effects models pooling results from both countries demonstrated a small yet significant decrease in the crude incidence rates (IRR = 0.998, *p* < 0.001). Standardized incidence rates decreased significantly in both countries (France: −1.6% per year, *p* < 0.001; Sweden: −0.6% per year, *p* < 0.001). The trends in crude and standardized incidence rates were consistent between countries during the overlapping study period (2009–2018) (eTable 2).

#### MS

Crude incidence rates were higher in Sweden (11.4 [95% CI 10.7–12.1]) than in France (7.79 [7.57–8.00]); the same pattern was noted for standardized incidence rates (Sweden: 11.2 [10.3–12.2]; France: 7.94 [7.70–8.19]), per 100,000 person-years (Table 2). In Sweden, crude incidence rates declined from 11.2 per 100,000 person-years in 2006 to 9.7 per 100,000 person-years in 2016 (IRR per year = 0.985, 95% CI 0.979–0.992) (Table 2, Figure 2, eFigure 5). In France, a modest decline was also noted, from 8.2 cases in 2011 to 7.2 cases in 2022 per 100,000 person-years (IRR = 0.993, 95% CI 0.991–0.995). The mixed-effects models indicated a modest but significant decline in crude incidence rates (IRR = 0.9898, 95% CI 0.9824–0.9973) and standardized incidence rates (IRR = 0.9928, 95% CI 0.9849–1.0008) (Table 2). The trends in incidence rates were consistent between countries during the overlapping study period except for the standardized incidence rates (Sweden: IRR = 0.987 [0.977–0.997], *p* = 0.011, France: IRR = 0.999 [0.994–1.003], *p* = 0.5) (eTable 2).

#### MNDs

Crude incidence rates were 4.07 (95% CI 3.64–4.50) in Sweden and 3.23 (3.10–3.37) in France, whereas standardized incidence rates were 3.90 (95% CI 3.46–4.33) in Sweden and 3.05 (95% CI 2.91–3.19) in France (Table 2). In Sweden, the crude incidence rates increased from 3.77 in 2003 to 4.47 in 2016 per 100,000 person-years, indicating an annual increase of 2.1% (*p* < 0.001) (Table 2, Figure 2, eFigure 5). In France, the crude incidence rates increased from 2.99 in 2011 to 3.43 in 2019 per 100,000 person-years, reflecting an annual rise of 1.5% (*p* < 0.001). Standardized incidence rates demonstrated an annual increase of 1.4% (*p* = 0.005) in Sweden and an annual increase of 0.5% in France (*p* = 0.078). Mixed-effects models confirmed the increasing trend for both the crude (IRR = 1.018 [1.013–1.022]) and standardized (IRR = 1.009 [1.001–1.017]) incidence rates. The trends in crude incidence rates were consistent between countries during the overlapping study period, although the rise in crude incidence was no longer significant in Sweden (IRR = 1.009 [0.993–1.025]). Standardized incidence rates were consistent across countries (Sweden: IRR = 1.000 [0.984–1.016], *p* = 0.9, France: IRR = 1.004 [0.998–1.011], *p* = 0.2) (eTable 2).

### Age at Diagnosis

**Figure 3:**
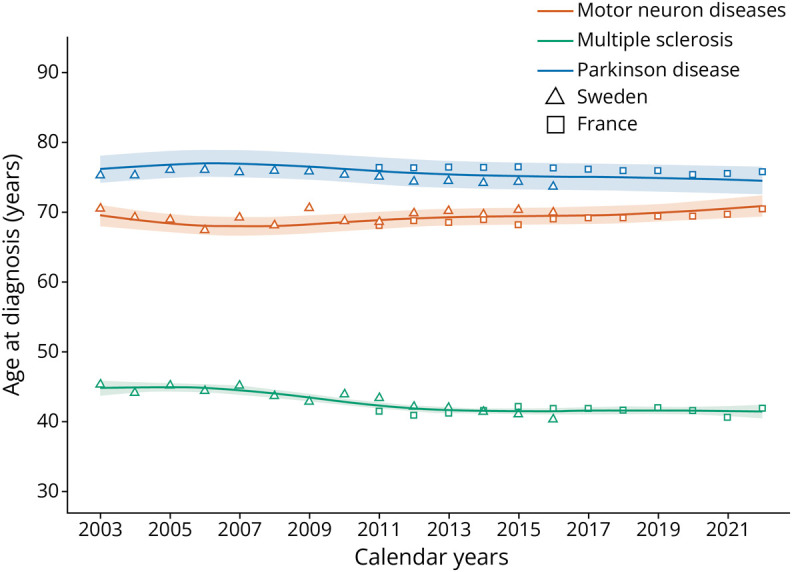
Secular Trends in Age at Incidence in Sweden and France (2003–2022) Age curves were estimated using single calendar year data points and the predictions of the population effect of nonlinear regression mixed-effects models. We assumed a nonlinear relationship between time and incidence, using restricted cubic splines interpolation.

The median age at diagnosis was also comparable between countries: 75.1 years in Sweden and 76.1 years in France for PD, 43.2 years in Sweden and 41.6 years in France for MS, and 69.4 years in Sweden and 69.1 years in France for MND (Table 1). This median age remained generally stable over time in both countries (Figure 3), with the notable exception of MS in Sweden, where it decreased by 5.0 years between 2003 and 2016. In Sweden, the median (interquartile range) age at diagnosis in 2003 was 45.3 (35.3–55.7), whereas it was 40.3 (30.0–51.7) in 2016.

### Life Expectancy at Diagnosis

**Figure 4:**
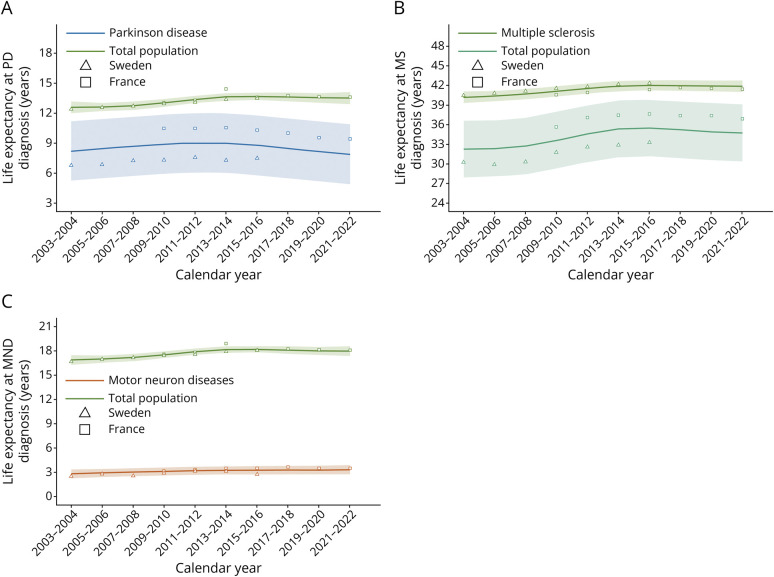
Secular Trends in Life Expectancy at Diagnosis of Patients With (A) PD, (B) MS, and (C) MNDs Compared With the General Population We assumed a nonlinear relationship between life expectancy and calendar time. MNDs = motor neuron diseases; MS = multiple sclerosis; PD = Parkinson disease.

When diagnosed in the years 2013–2014, life expectancy at PD diagnosis (median age: 75 years) was 7.5 years in Sweden, 10.9 years in France, and 9.0 years in the pooled analysis. In the reference population, life expectancy at the corresponding age was 13.3 years in Sweden and 13.9 years in France (Figure 4A, eTable 3). Over the entire study period (2003–2022) of the pooled analysis, life expectancy at PD diagnosis remained relatively stable (−0.21 months per calendar year, *p* = 0.5) although the life expectancy of the reference population increased by 0.87 months per calendar year (*p* = 0.005) (eTable 3). Life expectancy at diagnosis increased significantly between 2003 and 2013 (+0.95 months per calendar year, *p* < 0.001), but decreased between 2013 and 2022 (−1.20 months per calendar year, *p* = 0.002), in the pooled analysis.

Life expectancy at MS diagnosis (median age: 42 years) was 33.2 years in Sweden (vs 42.2 years in the reference population) and 37.6 years in France (vs 41.6 years in the reference population) in 2013–2014. Over the entire study period in the pooled analysis, life expectancy at MS diagnosis increased significantly (+2.35 months per calendar year, *p* < 0.001), that is, faster than the increase in life expectancy in the reference population (+1.32, *p* < 0.001) (Figure 4B, eTable 3).

Life expectancy at MND diagnosis (median age: 69 years) was 3.1 years in Sweden and 3.5 years in France in 2013–2014. Life expectancy at MND diagnosis showed a slight but statistically significant increase over time, with a gain of +0.34 months per calendar year (*p* = 0.014), in the pooled analysis (Figure 4C, eTable 3).

## Discussion

This study leveraged 2 nationwide, population-based European cohorts to provide a comprehensive analysis of temporal trends in the burden of PD, MS, and MNDs over the past 2 decades. Our headline findings confirm a rising prevalence of all 3 NDDs in both countries. To explain this increasing prevalence, our results highlight 3 key points. First, age at diagnosis remained relatively stable across both countries and all 3 diseases, suggesting that earlier detection is unlikely to account for the observed trends. The only exception was MS in the early 2000s in Sweden, coinciding with expanded access to magnetic resonance imaging. Second, while crude incidence rates remained nearly stable for PD and MS—and even declined slightly for PD after age standardization—both crude and standardized incidence rates of MND increased consistently in the pooled analyses. This pattern indicates that factors beyond demographic aging may be driving the rising incidence of MND. Third, increases in life expectancy at diagnosis largely explain the growing prevalence of MS. By contrast, the deceleration in life expectancy gains among patients with PD in the most recent decade (2013–2022), compared with the previous one (2003–2013), may underlie the recent stabilization in PD prevalence. Finally, because there was no clear gain in life expectancy at diagnosis for MND, the increase in its prevalence seems to be primarily driven by a genuine rise in incidence.

Prevalence of PD is rising globally.^21,22^ In our study, crude incidence rates of PD remained stable, whereas age-standardized and sex-standardized incidence rates declined during the study period, suggesting a potential real decrease in PD incidence. Recent projections have forecast a rising burden of PD based on demographic shifts^5,21,23^ and continued gains in survival of patients with PD, based on the historical trend of rising life expectancy in the general populations of France and Sweden (+0.25 months per year in France over the past 6 decades).^5^ However, we find that life expectancy at diagnosis of PD has plateaued, or even declined since 2010 in both Sweden and France, mirroring a broader stagnation relative to the improving life expectancy in the general population.^24^ This likely helps explain why PD prevalence has risen more slowly than some models predicted.^25^

The declining standardized incidence rates may reflect the changing profiles of risk factor exposure, or improved diagnostic specificity (e.g., fewer atypical parkinsonisms are misclassified as PD). Notably, the median age at PD diagnosis remained stable at approximately 75 years, suggesting little shift toward earlier detection over time, consistent with previous findings.^26,27^

We observed differences in the prevalence, incidence, and life expectancy at diagnosis of PD between Sweden and France. PD was more prevalent and had a higher incidence rate in France, whereas life expectancy at PD diagnosis was shorter in Sweden. Multiple reasons might underlie such differences. In Sweden, PD diagnoses are usually made in specialized inpatient or outpatient care, likely capturing more severe cases and contributing to lower prevalence and incidence and shorter survival. On the contrary, the French data set also included patients with PD managed entirely in primary care, even if this may involve evaluations by community neurologists, which may inflate estimates of prevalence and incidence rates by reduced diagnostic specificity. This diagnostic variability should, however, be less relevant for MS and MND, because specialist input is often required early on for these 2 conditions.

Our finding on the rising prevalence of MS corroborates and extends recent studies in developed Western countries, confirming an ongoing trend of increasing prevalence in line with improved survival.^7,28-30^ Incidence rates and age at diagnosis remained fairly stable in France throughout the study. However, in Sweden, a more marked decline in incidence rates was noted between 2001 and 2005, which may represent an artifact due to prevalent cases with rarer contacts with health care being misclassified as incident MS. This interpretation is supported by alternative data sources, such as the Swedish MS Register that shows a relatively stable MS incidence in 2000–2010, ranging from 10 to 12 per 100,000 person-years.^31^ To reduce this potential bias from using administrative registry data lacking precise information on the date of diagnosis, which is less relevant with PD and MND that typically involve more frequent follow-ups, we, therefore, opted for a 5-year washout period to capture true incident cases in subsequent analyses.

In addition, although revisions to the McDonald diagnostic criteria (in 2001, 2005, 2010, and 2017)^32-35^ have been made to allow for an earlier diagnosis of MS with preserved specificity and sensitivity because of incorporation of imaging and fluid biomarker data, they have not been paralleled by a sustained increase in MS incidence.^31^ Our findings, therefore, support the growing consensus that while regional differences in incidence trends may still exist,^36^ the dominant factor behind a rising prevalence of MS is improved survival rather than increased incidence.^37^ Arguably, this may bear witness to the importance of improved access to disease-modulatory therapies,^38^ which so far lack for PD and MND.

MND prevalence has increased, primarily due to population aging, as we observed a significant upward trend in crude incidence in France and Sweden.^25,39^ This trend was not entirely mitigated by age and sex standardization, however, suggesting that factors beyond aging, such as environmental exposures or health care access, may also play a role. Unlike other NDDs, the life expectancy at diagnosis of MND has remained stable over the past 2 decades, reflecting the limited impact of available treatments on disease progression.^40,41^

MND is particularly well suited for studies using national health registers because of its rapid progression and the need for hospital-based care, ensuring reliable diagnostic data. The consistency in age at diagnosis and life expectancy at diagnosis between Sweden and France, which differed more with MS or PD, supports the robustness of case identification and cross-country comparisons. The higher incidence observed in Sweden compared with France may reflect true epidemiologic differences rather than data artifacts. In fact, a recent Swedish study showed a north-south gradient in MND incidence,^13^ similar to patterns seen in MS.^42^ However, to what degree a similar geographic gradient also exists in Europe as a whole remains to be explored.

This study leverages nationwide health registers with population-wide coverage in 2 European countries, enabling robust analyses of long-term trends in large and representative cohorts. We selected PD, MS, and MND because they share a predominant motor phenotype, are among the most prevalent neurodegenerative disorders after Alzheimer disease, and, crucially, have well-defined, traceable care pathways in national health registers, including characteristic diagnostic codes, long-term disease status, specialist care, and specific reimbursed treatments, which together enable reliable and comparable case identification across both countries. A major strength is the use of truly population-based data, which captures the full range of disease severity, comorbidities, and age groups, different from clinical cohorts that are often biased toward younger or more severe patients. However, our study also has limitations. First, the generalizability of our results warrants careful consideration. Sweden and France are high-income European countries with universal health care coverage and well-established diagnostic and therapeutic infrastructures for NDDs. The stable PD incidence observed may partly reflect progress in reducing known risk factors (e.g., occupational exposures and pesticide regulations), which may not extend to countries with different environmental policies or exposure patterns. For MS, the survival improvements we observed are likely driven by disease-modifying therapies that are expensive and not universally available, limiting the applicability of our findings to lower resource settings. For MND, a north-south gradient in incidence has been previously reported in Sweden; however, whether our findings are representative of the entire European population or other populations remains unknown. Caution is, therefore, warranted when extrapolating these findings to other geographic or health care contexts, and similar studies in diverse settings are needed to assess the global applicability of these trends.

Despite efforts to standardize analyses, we observed notable differences in the incidence rates of NDDs: more incident cases of MS and MND were identified in Sweden, while PD was more frequently diagnosed in France. These variations could reflect true differences in disease risk between countries due to genetic (frequency of known risk alleles such as HLA-DRB1*15:01 for MS, LRRK2 for PD, and C9orf72 for MND), environmental, or other contextual factors. However, they may also be influenced by database-specific characteristics, such as differences in coding practices, access to specialist care and diagnostic services, or the criteria used to identify cases. The data sets did not provide precise information on the clinical onset of disease, limiting our ability to evaluate diagnostic delays or prodromal periods. Case identification relied on diagnostic codes in Sweden and on prescriptions and diagnostic codes in France, which may reduce specificity, particularly in the absence of confirmatory information. The study periods in Sweden and France were not entirely overlapping, which may have influenced comparisons, especially given temporal trends in disease recognition or coding. The washout periods applied, 2 years for PD and MND and 5 years for MS, were determined through visual inspection of the incidence curves, selecting the first year in which the incidence displayed a stable, monotonic pattern as the start of the study period to reduce the influence of prevalent cases. This approach required a trade-off between retaining sufficient data to observe meaningful temporal trends and removing individuals with preexisting disease. However, because the true underlying incidence trajectory is unknown, relying on apparent monotonicity may introduce misclassification, which represents a limitation of the study.^43^ Finally, the French study period included the COVID-19 pandemic, which may have influenced mortality patterns through both direct and indirect effects of the pandemic on health care access and vulnerable populations.

Our study confirms that the prevalence of MS, PD, and MND continues to rise, although the patterns in incidence and survival trends seem to differ between diseases. While improved survival—both disease-specific and as a byproduct of broader advances in the management of other chronic or systemic conditions—may have contributed to the rising prevalence of PD and MS, incidence trends remain heterogeneous across diseases. Leveraging large-scale health registries for multinational studies will provide deeper insights into the evolving epidemiology of NDDs and inform targeted public health interventions.

## Glossary

COVID-19: coronavirus disease 2019
GDPR: General Data Protection Regulation
ICD-19: International Classification of Diseases, Ninth Revision
ICD-10: International Classification of Diseases, Tenth Revision
IRR: incidence rate ratio
MND: motor neuron disease
MS: multiple sclerosis
NDD: neurodegenerative disease
PD: Parkinson disease
PR: prevalence ratio
SNDS: French National Health Data System
SNPR: Swedish National Patient Register
